# Sensor Access to the Cellular Microenvironment Using the Sensing Cell Culture Flask

**DOI:** 10.3390/bios8020044

**Published:** 2018-04-26

**Authors:** Jochen Kieninger, Yaara Tamari, Barbara Enderle, Gerhard Jobst, Joe A. Sandvik, Erik O. Pettersen, Gerald A. Urban

**Affiliations:** 1 Laboratory for Sensors, IMTEK—Department of Microsystems Engineering, University of Freiburg, Georges-Köhler Allee 103, D-79110 Freiburg, Germany; yaara.tamari@gmail.com (Y.T.); barbara.enderle@imtek.uni-freiburg.de (B.E.); urban@imtek.de (G.A.U.); 2 Jobst Technologies GmbH, Engesserstraße 4b, D-79108 Freiburg, Germany; gj@jobst-technologies.com; 3 Department of Physics, University of Oslo, 1048 Blindern, N-0316 Oslo, Norway; j.a.sandvik@fys.uio.no (J.A.S.); e.o.pettersen@fys.uio.no (E.O.P.)

**Keywords:** cell culture monitoring, microsensor, oxygen, pH, hypoxia

## Abstract

The Sensing Cell Culture Flask (SCCF) is a cell culture monitoring system accessing the cellular microenvironment in 2D cell culture using electrochemical microsensors. The system is based on microfabricated sensor chips embedded in standard cell culture flasks. Ideally, the sensor chips could be equipped with any electrochemical sensor. Its transparency allows optical inspection of the cells during measurement. The surface of the sensor chip is in-plane with the flask surface allowing undisturbed cell growth on the sensor chip. A custom developed rack system allows easy usage of multiple flasks in parallel within an incubator. The presented data demonstrates the application of the SCCF with brain tumor (T98G) and breast cancer (T-47D) cells. Amperometric oxygen sensors were used to monitor cellular respiration with different incubation conditions. Cellular acidification was accessed with potentiometric pH sensors using electrodeposited iridium oxide films. The system itself provides the foundation for electrochemical monitoring systems in 3D cell culture.

## 1. Introduction

Behavior of cells or their response to specific molecules can be studied by isolating cells from organisms and culturing in an artificial environment. While cells have been kept alive in vitro and researched for more than a century, the monitoring of cellular microenvironment is lagging behind the progress in cell culturing techniques. This is important because cell culture monitoring of metabolic parameters is an essential tool to ensure reproducible culture conditions. Furthermore, the continuous measurement of concentrations of small molecules provides biologists with the ability to gain new insights into the transient nature of many processes researched in cell culture.

The environment for cells cultivated in vitro is far away from the physiological case. The usage of microfluidic devices for the investigation of cellular responses to cyclic stimuli deviates even more from the natural situation. Early cell culture monitoring systems followed this dynamic approach. The main focus of those systems was on screening applications in cell culture. One of the first chip-based cell culture monitoring systems of this type was based on the light-addressable potentiometric sensor (LAPS) [1,2]. These works led at the beginning of the 1990s to the commercial Cytsensor Microphysiometer system allowing the measurement of cellular acidification rates with cells in a microfluidic system [3,4]. The system was further enhanced with oxygen, glucose and lactate sensors [5,6] in order to enable multi-parameter measurements. Similar approaches of cultivating cells along with a microfluidic system and the monitoring of acidification and respiration by ISFETs, potentiometric or amperometric sensors [7,8,9,10] and biosensors [11] were presented. The main application of those systems is in the field of drug screening [12].

In parallel, the need to monitor metabolic parameters in static cell culture conditions became obvious. Especially in 2D cell culture, concentration gradients along the medium height can be observed. Those gradients may cause completely different conditions in the cellular microenvironment compared to the bulk of the cell culture medium [13]. This is most critical in the case of oxygen as demonstrated by measuring the concentration gradient along the medium height in a stagnant culture [14]. Optical systems have been developed and used for cell culture monitoring application, mainly for oxygen and pH measurements [15,16,17]. These systems allow metabolic monitoring in static culture in order to control culture conditions as well as to investigate transient effects further. However, optical systems have, compared to electrochemical approaches, fewer prospects for the combination of multiple sensors measuring different parameters within the same compartment.

Still there has been a lack of sensors combining the major metabolic parameters (such as oxygen, pH, glucose, lactate), as well as parameters of interest in a specific field (such as reactive oxygen species, nitric oxide or neurotransmitters) with an easy-to-use platform. We therefore introduced the concept of the Sensing Cell Culture Flask (SCCF) in 2007 [18]. The SCCF platform was designed as a generic cell culture monitoring system allowing the integration of any electrochemical sensor. The distinct feature is the usage of a standard cell culture flask in order to keep the biological routine as far as possible undisturbed by the presence of the sensors and instrumentation. At present, sensors for oxygen, pH, glucose, lactate, nitric oxide, and superoxide have been implemented in the SCCF system [19,20,21,22].

In this paper we describe the fabrication of the SCCF sensor chips, the system design and its application in cell culture experiments with oxygen and pH sensors as typical representatives for amperometric and potentiometric measurements. A detailed study on the pericellular oxygen monitoring with the SCCF system was published in [21].

## 2. Materials and Methods

### 2.1. Sensor Chip Fabrication

**Figure 1:**
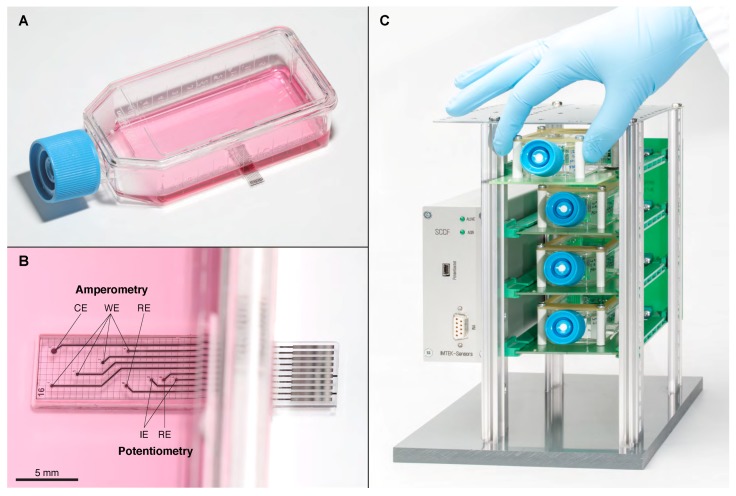
(**A**) Sensing Cell Culture Flask (SCCF) sensor chip embedded in the bottom of a cell culture flask; (**B**) detail view of the sensor chip with the regions for amperometry and potentiometry (counter electrode (CE), working electrode (WE), reference electrode (RE), indicator electrode (IE)); (**C**) rack system with four slots occupied by the reusable frames comprising single-use SCCFs; the casing on the left contains the multiplexer and potentiostat.

The SCCF sensor chips (Figure 1A) were fabricated using 100 mm borosilicate glass wafers with 500 μm thickness in order to allow optical inspection of cells through the sensor chip. Prior to metal deposition, a 500 nm silicon nitride layer was deposited by plasma enhanced vapor deposition (PECVD). The metal layers were 50 nm titanium, 100 nm platinum and 20 nm titanium coated by vapor deposition and structured by a lift-off process with an image reversal photoresist.

For passivation, a 800 nm silicon nitride layer followed by a 200 nm silicon oxide layer was deposited by PECVD. This layer is the surface layer exposed to the cells. The passivation layer stack was structured using reactive ion etching, which also removed the titanium from on top of electrodes and connection pads exposing the platinum. All electrode openings were 200 μm in diameter except for the counter electrode with 400 μm.

The Ag/AgCl reference electrodes were formed on wafer-level by electrodeposition of 10 μm silver in a cyanide-based plating bath (Arguna S, Umicore, Hanau, Germany) at room temperature. Current density during deposition was −16 mA cm^−2^ for 600 s. Subsequently the silver was partially electrochemically converted into silver chloride in a 0.1 M KCl solution at 1.6 mA cm^−2^ for 1200 s.

pH electrodes were obtained by electrodeposition of iridium oxide on chip-level. The iridium oxide was formed using a deposition solution with 1.5 g/L IrCl_4_·H_2_O, 10 mL/L H_2_O_2_ (30 wt %) and 5 g/L (COOH)_2_·2H_2_O in water. The solution was prepared according to [23] with K_2_CO_3_ to adjust the pH. To achieve well-adhering layers on thin-film platinum electrodes a two-step procedure was applied: By cycling the voltage between 0.18 and 1.48 V_RHE_ (potential in terms of Reversible Hydrogen Electrode) with a scan rate of 0.1 V/s for 5 cycles an adhesion layer was formed. Subsequently, the iridium oxide was deposited applying a constant potential of 1.43 V_RHE_ for 300 s.

**Figure 2:**
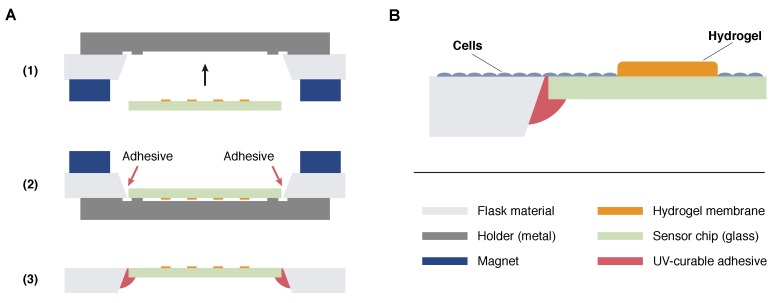
(**A**) Hybrid integration of the sensor chips in a milled opening in the bottom of a cell culture flask. A metallic holder was brought inside the flask and fixed with magnets from outside (1). The flask was flipped, sensor chips were positioned in a opening milled into the bottom, and UV-curable adhesive was applied (2). After curing, the magnets were removed and the holder released through the neck of the flask resulting in a positioned chip with its surface in-plane with the flask surface (3); (**B**) Schematic representation of the cell layer. Cells are supposed to settle on the flask and chip surface but not on the hydrogel membranes covering the sensor electrodes.

All electrodes were covered with a pHEMA-based hydrogel. The hydrogel precursor solution consisted of 28% HEMA, 26% pHEMA, 42% ethylene glycol, 3% TEGDMA, and 1% Irgacure 651 in water. The solution was dispensed onto the electrodes by a Computer Numerical Control (CNC) dispenser and cured in a nitrogen atmosphere by UV light exposure. The dispenser was a custom-modified 3-axis CNC machine (CPM 3020, Isel, Eichenzell, Germany) operated with the software VisualCNC (Jobst Technologies, Freiburg, Germany). The hydrogel membranes act as a diffusion barrier in case of the oxygen sensor working electrodes and prevents direct contact of the cells with the electrodes in all cases. Normally the cells are supposed to settle next to the hydrogel as illustrated in Figure 2B. Only in rare cases a cell was observed on the hydrogel membrane.

The wafers were diced into 5.5 mm × 24 mm sized sensor chips resulting in 30 chips per wafer. The sensor chips were integrated into the bottom of cell culture flasks so that the surface of the sensor chip was in-plane with the flask surface avoiding any steps in the area where cells are supposed to settle (Figure 1A,B). Therefore, an opening with slanted edges was milled into the bottom of a conventional tissue culture flask, and the sensor chip was aligned to ensure a continuous surface of cell growth area. The chip was fixed, and the gap was sealed with the UV-curable adhesive Loctite 3201 (Henkel, Düsseldorf, Germany) and hardened by UV light exposure. These hybrid integration steps are illustrated in Figure 2A. All cell culture experiments described in this work were done with SCCFs based on BD Falcon PRIMARIA tissue culture flasks, 25 cm^2^, canted neck (Becton Dickinson Labware, Franklin Lakes, NJ, USA).

### 2.2. Instrumentation

**Figure 3:**
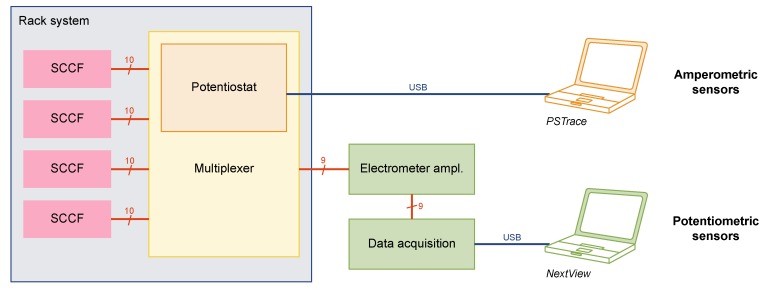
Block diagram of the SCCF instrumentation: Four flasks can be inserted into the rack system. Amperometric measurements are realized by a single-channel potentiostat integrated into a custom-made multiplexer. Potentiometric measurements can run continuously by a multichannel electrometer amplifier and data acquisition device.

The concept of the SCCF system is based on single-use cell culture flasks with embedded sensor chips (Figure 1A,B), a reusable frame to protect the sensor chip and establish the electrical connections, and a rack system (Figure 1C). The frames were based on printed circuit boards ensuring mechanical stability while retaining the possibility for optical inspections of the cells from below using an inverted microscope. Four of those frames could be inserted into a custom-designed rack system comprising a multiplexer unit for the amperometric sensors and the wiring for the potentiometric sensor points. A block diagram of the instrumentation is shown in Figure 3.

The amperometric sensors were controlled and measured using the EmStat OEM module (PalmSens, Houten, the Netherlands) as potentiostat and the related PSTrace software. Up to 16 working electrodes could be sequentially accessed by a custom-made multiplexer, which was controlled through digital outputs of the potentiostat using the scripting functionality of the PSTrace software. The switching was realized using mechanical relays to avoid any leakage current.

A custom-made electrometer amplifier based on the instrumentation amplifier INA 116P (Burr Brown, Tucson, AZ, USA; nowadays Texas Instruments, Dallas, TX, USA) was used for the potentiometric measurements. The device features eight channels with common reference electrode. Data acquisition of the potentiometric sensors was done using the meM-ADDA device (BMC Messsysteme, Maisach, Germany) in combination with the related NextView software.

The rack system allowed the operation of four SCCFs in parallel with four amperometric and two potentiometric sensor points in each flask, thus adding up to 16 amperometric and eight potentiometric measurement channels. Amperometric sensors were read sequentially, while potentiometric sensors were read truly parallel.

### 2.3. Oxygen Measurement

Optimized oxygen sensors are needed in order to obtain stable sensor readings in cell culture media for several days without the need for recalibration. Stability can be achieved by separation of the sensor electrolyte from the measurement medium by a gas-permeable membrane (such as in the case of Clark-type sensors) or specific cleaning protocols as described below in order to minimize electrode blocking and subsequent loss in sensitivity. To keep the fabrication steps simple and the SCCF chip technology generic we opted for the second approach. The oxygen sensors were direct amperometric sensors (the measurement analyte is the sensor electrolyte) operated by a chronoamperometric pulse protocol with three steps. Each cycle is followed a longer off-time in order to minimize the inherent overall analyte consumption of the oxygen sensor.

All potentials were referred to the on-chip silver/silver chloride electrode. We report the potentials in terms of the Reversible Hydrogen Electrode (RHE) scale taking into account the pH. Potentials vs. RHE were obtained by adding 0.288 V (standard potential of Ag/AgCl at 37 ${}^{{}^{\circ}}C$ in 0.1 M Cl^−^) and 0.455 V (reflecting pH 7.4 at 37 ${}^{{}^{\circ}}C$) to the potential referred to the on-chip reference electrode. In the first step (1.54 V_RHE_, 4 s duration) the working electrode was polarized in the regime of platinum oxide formation. Subsequently, the formed platinum oxide was reduced in the second step (0.34 V_RHE_, 3 s). The sensor readings were taken at the end of the third step (0.44 V_RHE_, 3 s). One measurement cycle took 10 s per electrode. During the off-time the multiplexer connected other working electrodes to the potentiostat. The off-time was adjusted to obtain an overall acquisition interval of 5 min. Such chronoamperometric pulse protocols are described in more detail in [18].

We generally report the concentration of dissolved oxygen in M (mol/L). To be consistent with the majority of biological literature, we scaled the results from cell culture monitoring to “% O_2_”. 1% O_2_ corresponds to 9.5 μM dissolved oxygen concentration at 37 ${}^{{}^{\circ}}C$ adjusted for salinity of the cell culture medium. This scale can be understood as concentration of dissolved oxygen equilibrated to a gas phase which contains the corresponding fraction of oxygen at normal pressure. For oxygen monitoring with cells, sensitivities of each sensor electrode were calibrated individually in cell culture medium at 4% O_2_ or 20% O_2_ prior to seeding of the cells. Offsets were obtained by batch-calibration. All oxygen sensor results with cells were reported as mean value of data from two sensor points on the sensor chip.

### 2.4. pH Measurement

pH electrodes were characterized at 37 ${}^{{}^{\circ}}C$ and room temperature in phosphate buffered saline (PBS) comprising 0.1 M NaCl. Different ratios of Na_2_HPO_4_ and NaH_2_PO_4_ in concentrations summing up to 0.1 M were used to obtain different values in the range pH 6–8. Sensitivities used during the cell culture measurements were obtained from calibration in buffer solution, while the offset was adjusted to the value obtained in cell culture medium.

For the presented pH measurements with cells an electrode which is normally dedicated to amperometry (leftmost WE in Figure 1B) was coated with iridium oxide and operated as indicator electrode during potentiometry. In this way it was possible to obtain data from three pH sensor electrodes within the same cell culture flask.

### 2.5. Cell Culture Experiments

Two cell lines were used in this work: T-47D breast cancer cells [24], which show highly active metabolism, and T98G brain tumor cells [25] derived from a human gliablastoma multiforme tumor. T-47D cells were used for the pH, and T98G cells for the oxygen measurements. Both cell lines were grown as monolayer cultures in RPMI 1640 medium (Gibco, Rockville, MD, USA), supplemented with 10% foetal calf serum (Gibco), 200 units/L insulin and penicillin/streptomycin (Gibco). In case of T-47D cells the medium was changed during the experiment to a weakly buffered RPMI 1640 medium. In comparison to normal RPMI 1640, the concentration of NaHCO_3_ was lowered from 24 to 6 mM while keeping all other salts at the same concentration.

$0.5\times 10^{6}$ T98G cells per flask were seeded for the oxygen monitoring experiments. For the pH monitoring $3\times 10^{6}$ T-47D cells per flask were seeded. Cells for the pH measurement as well as the oxygen measurements under atmospheric oxygen conditions were cultured in a special walk-in incubator room at 37 ${}^{{}^{\circ}}C$. The flasks were equipped with tight caps encapsulating an atmosphere containing 20% O_2_ and 5% CO_2_. Cells for the oxygen measurements under hypoxic incubation condition were seeded in air before the flasks were transferred into an INVIVO2 400 hypoxic workstation (Ruskinn Technology, Bridgend, UK) with an atmosphere containing 4% O_2_ and 5% CO_2_. These SCCFs were equipped with vented filter caps allowing the atmosphere in the flask to equilibrate with that of the hypoxia workstation.

Although the clean-room fabrication process results in a low biological burden in the SCCFs, all flasks were sterilized with 1000 Gy, 4 MeV electron beam from a clinical linear accelerator, Elekta Synergy (Elekta Instrument, Stockholm, Sweden) before seeding of the cells.

## 3. Results and Discussion

### 3.1. SCCF System

The Sensing Cell Culture Flask (SCCF) system was developed to disturb routine work flow in cell culture laboratories as little as possible. The transparent sensor chips were embedded in the bottom of standard tissue culture flasks. During the development of the design it became evident that it is essential to have the surface of the sensor chip at the same level as the flask surface. This ensures similar diffusion properties towards the cells settling on the sensor chip and on the flask material. Cell density evaluation and clonogenic survival rate of T-47D cells showed no significant difference in cell growth on the flask material compared to the sensor chip surface [21].

The present system allows measuring with four cell culture flasks in parallel. The system can be easily scaled up with the same requirement of footprint in the incubator. The multiplexing during amperometric measurements was not a limiting factor because of the slow cellular responses.

### 3.2. Oxygen Sensors

**Figure 4:**
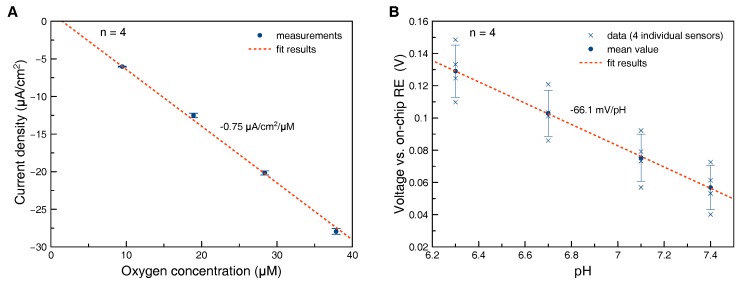
Calibration plot oxygen sensor (**A**): Sensors were calibrated at 37 ${}^{{}^{\circ}}C$ in cell culture medium equilibrated to different oxygen content and 5% CO_2_ in the gas phase. Error bars represent the standard deviation of measurements from four individual electrodes fabricated in the same batch, calibration plot pH sensor (**B**): Sensors were calibrated after sterilization in buffer solution at 25 ${}^{{}^{\circ}}C$. Both mean value and data points from four individual electrodes were plotted. Please note that the large error bars (standard deviation) are caused by variation of the offset rather than of the sensitivity.

Oxygen sensors were characterized with respect to sensitivity and stability in cell culture medium at 37 ${}^{{}^{\circ}}C$. Sensitivity was found to be separate-uncertainty (−0.75±0.02) μA/cm^2^/μM (n=4) (Figure 4A). The variation in sensitivity between individual electrodes produced in the same batch was less than 3%, while a larger variation in sensitivities between different batches was observed. It is assumed that the variation in sensitivity is caused by different geometries of the hydrogel acting as diffusion limiting membrane. After a run-in phase of one day the drift rate was found to be less than 0.1 μM/day, corresponding to 0.01% O_2_/day measured at 4% O_2_ in cell culture medium at 37 ${}^{{}^{\circ}}C$.

### 3.3. pH Sensors

The sensitivity of the bare iridium oxide films was measured in buffer solutions in the range of pH 6–8 at 37 ${}^{{}^{\circ}}C$ after continuous immersion in the solution at pH 7.4 for three days at 37 ${}^{{}^{\circ}}C$. The sensitivity was found to be separate-uncertainty (−77±3) mV/pH (n=5) corresponding to −74 mV/pH at 25 ${}^{{}^{\circ}}C$. This indicates that the iridium oxide films are predominantly anhydrous with some influence of hydrous iridium oxide following the model summarized in [26]. After coverage of the iridium oxide electrode with a pHEMA membrane and sterilization, the sensitivities were found to be separate-uncertainty (−66±2) mV/pH (n=4) at room temperature (Figure 4B). We speculate that this loss in sensitivity could be caused by partial conversion of hydrous to anhydrous iridium oxide caused by the irradiation during sterilization.

The offsets were found to be separate-uncertainty (545±29) mV (n=4) at room temperature. In contrast to the variation of the sensitivity, the variation of the offset was quite large. Therefore, offset calibration was done for each individual sensor point. Sterilized sensors were immersed again in buffer solution at 37 ${}^{{}^{\circ}}C$ before cell culture monitoring. The pH sensor signal increased (run-in drift) for up to two days, followed by a phase of nearly constant signal. The drift rate after the run-in drift was less than 0.4 mpH/h, measured at pH 7.4.

### 3.4. Oxygen Measurement with T98G Cells

**Figure 5:**
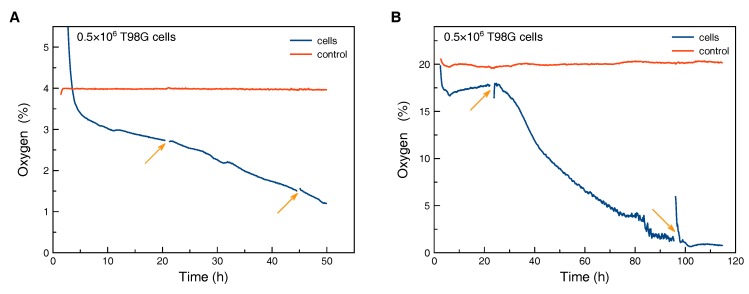
Monitoring of peri-cellular oxygen tension: The gas phase contained 4% O_2_ to model hypoxia (**A**) and 20% O_2_ (**B**). After several days of incubation the culture with high oxygen content in the gas phase also showed pericellular oxygen levels which are normally associated with hypoxia. The arrows indicate disconnection of the measurement and transfer of the flask to a microscopy stage for optical inspection of the cells. The control curve was measured in a SCCF without cells, which was kept in the respective incubation conditions.

Monitoring of the pericellular oxygen concentration was done at hypoxic conditions with 4% O_2_ (Figure 5A) and with 20% O_2_ in the gas phase (Figure 5B). The decrease of the pericellular oxygen concentration in both cases was caused by higher overall oxygen consumption. We assume that this decrease was mainly caused by cell proliferation, but a change in cellular respiration would have been possible as well.

The control flask without cells was kept in the hypoxia workstation for the duration of the hypoxic experiment (Figure 5A). Cells were seeded in a second flask in a flow bench at time zero and afterwards transferred to the hypoxia workstation. During the first hours a drop in oxygen concentration from values above those from the control flask to around 3% O_2_ was observed, caused by the exchange of the gas atmosphere in the flask. Afterwards, the oxygen concentration dropped continuously. Interruptions of the measurement and disturbance of oxygen diffusion profiles within the flask due to transfer to a microscopy stage within the hypoxia workstation (arrows in Figure 5A) did not change the slope of the oxygen curve.

For comparison, the same amount of cells was seeded and kept at 20% O_2_ in the gas phase (Figure 5B). We assume that the fluctuations in the reading from the control flask are mainly caused by temperature variations of the walk-in incubation room. After an initial drop the pericellular oxygen concentration in the flask with cells did stabilize at a plateau for roughly one day. We assume that the initial drop was caused by settlement of the cells and building-up of an oxygen gradient. The plateau indicates constant cell density and respiration. After the first transfer to a microscopy stage, a steeper decrease in pericellular oxygen concentration was observed. We speculate that the transfer and therefore strong oxygenation due to disturbance of the oxygen gradient within the cell culture medium might cause stronger proliferation or respiration. After more than three days, oxygen values were observed in the culture with 20% O_2_ in the gas phase which would normally be associated with hypoxic culture conditions. This clearly illustrates the possibly strong deviation between the preset oxygen content in the gas phase and pericellular concentration.

### 3.5. Acidification Measurements with T-47D Cells

**Figure 6:**
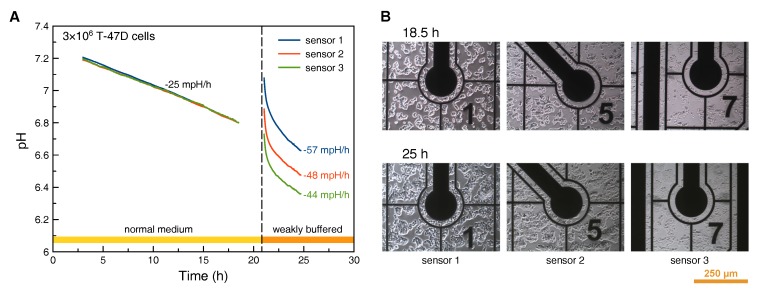
pH monitoring in high density cell culture: Sensor readings (**A**) from cells cultured in normal and weakly buffered medium; The cell images (**B**) were taken close to the three different pH sensor electrodes.

Acidification measurements were done sequentially in normal cell culture medium and weakly buffered medium (Figure 6A). After seeding of the high density cell culture, all three sensor points showed a steady, approximately linear acidification. The apparent acidification rate was −25 mpH/h; no significant difference between the readings from the three electrodes was observed. The observed acidification is sufficiently high compared to the electrode’s inherent drift rate (less than 0.4 mpH/h).

In contrast, after medium exchange to weakly buffered medium, the acidification appears non-linear, with a roughly twice as high rate in a quasi-linear region of the curve. With the reduced buffer strength, heterogeneity of the cell density is visible in the pH measurement. All three electrodes show different absolute values and acidification rates. The highest acidification rate (sensor 1) can be found in the case of highest cell density next to the sensor electrode. Accordingly, cell density next to the sensor electrode is lowest for the lowest acidification rate (sensor 3), see Figure 6B.

Application of the prescribed pH sensor electrodes with their inherent drift rate of less than 0.4 mpH/h makes them well suited for acidification measurements in high density cell culture or in combination with weakly buffered cell culture media. However, in case of low density culture and long culture periods without medium exchange, these types of iridium oxide pH electrodes need careful consideration of the expected acidification in comparison to the drift rate. The apparent acidification rate depends on ion strength of the pH buffer. Therefore, measurements in cell culture medium can provide relative acidification rates only.

## 4. Conclusions

The Sensing Cell Culture Flask (SCCF) is a cell culture monitoring system providing access to the microenvironment in 2D cell culture using electrochemical microsensors. It was shown that the approach of integrating microsensor chips enables the monitoring of the pericellular parameter with both potentiometric and amperometric sensors. The developed rack systems along with their instrumentation facilitate the combination of microsensor measurements with cell biology lab routines.

Oxygen measurements demonstrated the necessity of pericellular monitoring. Even with 20% O_2_ in the incubator gas phase, pericellular oxygen levels were found which are normally associated with hypoxia. pH measurements showed the possibility to access acidification rates in high density cell cultures with normal and weakly buffered cell culture medium.

While many recent biological works focus on 3D cell cultures, 2D cultures are still commonly used and information on the cellular microenvironment is needed. Additionally, the presented system can give important hints of the imperfect diffusion situation in 2D culture compared to 3D and therefore help to decide in which case a 3D cell culture is more appropriate [13]. Future work for the SCCF system will focus on the implementation of 3D cultures on the microsensor chip.
